# Factors predictive of the success of tuberculosis treatment: A systematic review with meta-analysis

**DOI:** 10.1371/journal.pone.0226507

**Published:** 2019-12-27

**Authors:** Ninfa Marlen Chaves Torres, Jecxy Julieth Quijano Rodríguez, Pablo Sebastián Porras Andrade, María Belen Arriaga, Eduardo Martins Netto

**Affiliations:** 1 Department of Medicine and Health, Federal University of Bahia, Salvador, Bahia, Brazil; 2 Department of Medicine, Nueva Granada Military University, Bogotá, D.C., Colombia; 3 Department of Biology, The University of Queensland, Brisbane, Australia; 4 Gonzalo Moniz Institute, Gonzalo Cruz Foundation, Salvador, Bahia, Brazil; 5 Department of Epidemiology, José Silveira Foundation, Salvador, Bahia, Brazil; Jamia Hamdard, INDIA

## Abstract

**Objective:**

To produce pooled estimates of the global results of tuberculosis (TB) treatment and analyze the predictive factors of successful TB treatment.

**Methods:**

Studies published between 2014 and 2019 that reported the results of the treatment of pulmonary TB and the factors that influenced these results. The quality of the studies was evaluated according to the Newcastle-Ottawa quality assessment scale. A random effects model was used to calculate the pooled odds ratio (OR) and 95% confidence interval (CI). This review was registered in the International Prospective Register of Systematic Reviews (PROSPERO) in February 2019 under number CRD42019121512.

**Results:**

A total of 151 studies met the criteria for inclusion in this review. The success rate for the treatment of drug-sensitive TB in adults was 80.1% (95% CI: 78.4–81.7). America had the lowest treatment success rate, 75.9% (95% CI: 73.8–77.9), and Oceania had the highest, 83.9% (95% CI: 75.2–91.0). In children, the success rate was 84.8% (95% CI: 77.7–90.7); in patients coinfected with HIV, it was 71.0% (95% CI: 63.7–77.8), in patients with multidrug-resistant TB, it was 58.4% (95% CI: 51.4–64.6), in patients with and extensively drug-resistant TB it was 27.1% (12.7–44.5). Patients with negative sputum smears two months after treatment were almost three times more likely to be successfully treated (OR 2.7; 1.5–4.8), whereas patients younger than 65 years (OR 2.0; 1.7–2.4), nondrinkers (OR 2.0; 1.6–2.4) and HIV-negative patients (OR 1.9; 1.6–2.5 3) were two times more likely to be successfully treated.

**Conclusion:**

The success of TB treatment at the global level was good, but was still below the defined threshold of 85%. Factors such as age, sex, alcohol consumption, smoking, lack of sputum conversion at two months of treatment and HIV affected the success of TB treatment.

## Introduction

Tuberculosis (TB) remains the leading global cause of death by a single infectious agent; it caused approximately 1.6 million deaths in 2017. An estimated 10 million people developed the disease, of whom 6.4 million (64%) were notified[1]. Additionally, of the 558,000 estimated cases of rifampicin- and isoniazid-resistant TB (multidrug-resistant TB—MDR-TB)/rifampin-resistant TB (RR-TB), a total of 139,114 people (87%) received the second-line regimen, and the proportion of MDR-TB cases with extensively drug-resistant TB (XDR-TB) defined as MDR-TB plus resistance to at least one drug in both of the two most important classes of medicines in an MDR-TB regimen: fluoroquinolones and second-line injectable agents (amikacin, capreomycin or kanamycin) was 8.5% (95% CI: 6.2–11%) [1].

The innumerable efforts to end the global TB epidemic have resulted in remarkable developments in research focused on multiple aspects of the disease. Unfortunately, we should rely on poor diagnostic, therapeutic, and preventive options. However, it is estimated that with the current strategies for TB control, the goals of reducing the number of deaths by 95%, reducing the incidence rate by 90% and increasing the cure rate of patients receiving first-line treatment to 90% between 2015 and 2035 will not be reached without intensifying research and development[2]. It is also necessary to strengthen health systems’ ability to detect cases early and to improve the quality of care, diagnosis and treatment of people with TB[3].

TB treatment coverage is one of the ten priority indicators for achieving the goals of the End TB Strategy, and it has increased from 51% in 2013 to 70% in 2017[2,4]. However, the treatment success rate has decreased from 86% in 2013 to 82% in 2016; in MDR-TB/RR-TB and XDR-TB cases, the success rate remains low: 55% and 34% in 2015[1]. This situation could be related to the limited evaluation of treatment outcomes in countries with limited resources and to the presence of factors that affect the outcome of TB treatment. Exhaustive estimates of TB treatment outcomes are needed to improve the programmatic management of TB. Therefore, this review with meta-analysis was performed to produce pooled estimates of global TB treatment outcomes and to analyze the predictive factors of successful TB treatment.

## Methods

This review was registered in the International Prospective Register of Systematic Reviews (PROSPERO) in February 2019 under number CRD42019121512.

### Search strategy

PubMed, Medline, Embase, ProQuest, Scopus and Scielo were searched for publications of the last 6 years that is, published between January 2014 and November 2019 that reported the results of treatment for pulmonary tuberculosis and the factors that influenced these results. We also searched other sources, such as Google and Google Scholar, and bibliographies to obtain additional references. Our search contained the following terms: tuberculosis, predictive factors, risk factors and treatment outcomes (tuberculosis AND (risk factors OR associated factors OR predictive factors OR characteristics) AND (treatment results OR treatment outcome OR successful treatment OR unsuccessful treatment OR unfavorable outcome OR (poverty OR poor)) AND tuberculosis treatment results) in English, Spanish and Portuguese. Trying to include as many publications as possible about our topic of interest. Approval from the ethics committee was not required.

### Data extraction and definitions

**Fig 1:**
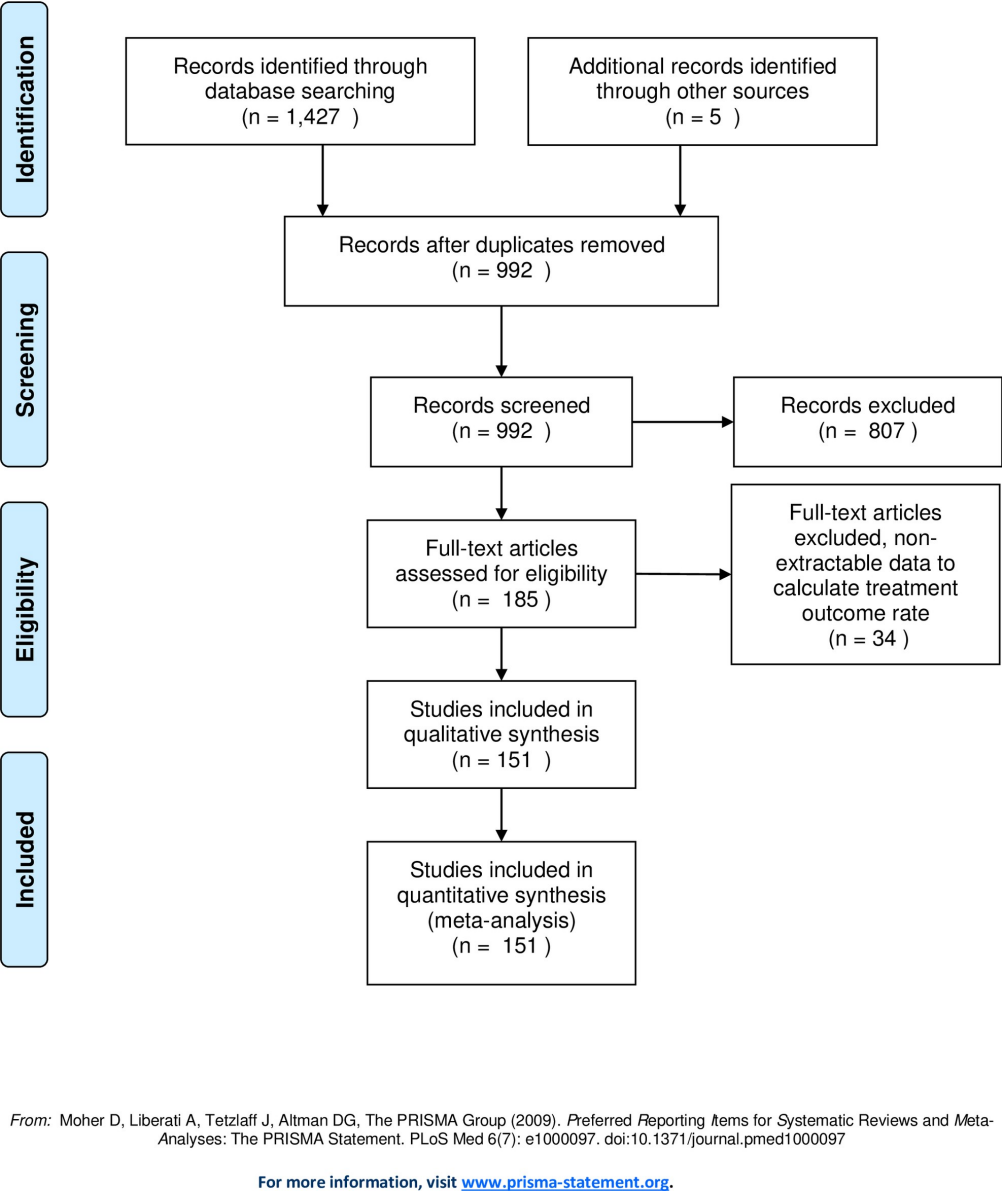
PRISMA flow chart indicating the result of literature search.

The step-by-step selection of the studies is described in Fig 1. All article titles and abstracts were evaluated by two investigators (JR and PP), including all the studies that reported quantitative measurements of the results of tuberculosis treatment, and these results were clearly described according to the WHO criteria. For cases of drug-sensitive TB, only studies that clearly described patients receiving the standard treatment for tuberculosis recommended by the WHO known as short-term treatment (6 months) that includes 4 drugs. Studies that reported exclusively on extrapulmonary tuberculosis and those that did not allow the adequate extraction of quantitative data were not included. The full text of articles identified as relevant by any of the reviewers was read.

To determine which full-text articles met the inclusion criteria, two investigators (JR and PP) reviewed all full-text articles, and a third investigator (NC) reviewed a random selection of studies. In cases of disagreement, the two investigators discussed the article until they agreed. One investigator (JR) extracted data from all included studies. The second investigator (NC) independently extracted all numerical data regarding the estimation of the main effect to validate the first review. If data from the same cohort were included in several articles, the article with the most complete data was included. For each included study, detailed information was collected on the design, publication year, country, study population, sample size, definition and measurement of treatment outcomes and associated factors.

### Validity assessment

The quality of the included studies was evaluated according to the Newcastle-Ottawa quality assessment scale[5]. It evaluates quality based on the content, design and ease of use of the data for meta-analysis. Two investigators (JR and PP) independently evaluated the quality of the studies, classifying each study as being of either good, acceptable or low quality.

### Statistical methods and data synthesis

TB treatment outcome measures were evaluated as the percentage of successful and unsuccessful results among all patients who initiated anti-TB therapy. The results of treatment were defined according to WHO criteria[6]. Successful outcomes were those in which patients met the definition of 'cure' or ‘treatment completion’. Unsuccessful outcomes were those in which patients met the definitions of death, default, failure or transfer. The subgroup analysis was performed by continent (Africa, America, Asia, Europe and Oceania), people living with HIV, children (1 to 15 years of age) and MDR/XDR-TB.

The associations of different variables, such as age (<65 years—66 years or older), sex (male—female), area of residence (rural—urban), type of case (new—previously treated), form of TB (pulmonary—extrapulmonary), alcohol consumption (yes—no), smoking (yes—no), HIV status (positive—negative), diabetes (yes—no), baseline sputum smear (positive—negative), and sputum smear microscopy two months after treatment (positive—negative), with the TB treatment outcome were measured. It was not possible to test the association from nutritional status, of educational level or socioeconomic status with TB treatment because these variables were evaluated differently in the studies depending on the country or region of origin. The influence of the health facility providing treatment on the results of TB treatment was not addressed in the studies included in this review.

Because most of the studies did not examine the association between these variables and successful treatment outcome as the main effect, unadjusted odds ratios (OR) and 95% confidence intervals (CI) were calculated as estimates of this association. After a table of results was created for each of the analyses, a random effects model was used to calculate pooled ORs and 95% CIs as there were high levels of heterogeneity in the study populations. Statistical heterogeneity was assessed using the I^2^ statistic. Publication bias was assessed using a funnel plot. All statistical analyses were performed in MedCalc® version 19.03.

## Results

As indicated in Fig 1, a total of 1,432 articles were identified. Of these, 992 were not duplicated; 807 of those were excluded after the title and abstract were evaluated, and 185 underwent a detailed review of the full text. A total of 151 studies with 1,550,449 patients with TB from 59 countries distributed among 5 continents met the criteria for inclusion in this review (Table 1).

**Table 1:** Distribution of patients with TB in the continents.

Continent	No. Countries	No. Studies	%	Pacientes	%
Africa	15	47	31.1	164,091	10.6
America	6	20	13.2	716,992	46.2
Asia	19	58	38.4	357,163	23.0
Europe	12	18	11.9	302,557	19.5
Oceania	5	5	3.3	6,926	0.4
Intercontinental	3	3	2.0	2,740	0.2
Total	60	151	100.0	1,550,469	100.0

In total, 95 of the 151 studies were retrospective cohort, 28 were cross-sectional, 25 were prospective cohort and 3 were case-control studies. Of the eligible studies, 91 reported treatment results in cases of TB in adults, 7 in children, 15 in patients coinfected with HIV and 38 in MDR/XDR-TB cases. These studies are detailed in Table 2.

**Table 2:** Characteristics of the included studies according to continent of origin and population studied.

Autor (Year)	Country	Study design	n	Success	%	Quality / newcastle
**TB studies in Africa**						
Adane K. et al (2018)[7]	Ethiopia	RC	422	395	93,6	L
Ali M. et al (2017)[8]	Somalia	CS	385	315	81,8	L
Amante T. et al (2015)[9]	Ethiopia	CC	976	646	66,2	L
Budgell E. et al (2016)[10]	South Africa	RC	544	394	72,4	A
Chidubem L. et al (2016)[11]	Nigeria	RC	555	479	86,3	L
Ejeta E. et al (2018)[12]	Ethiopia	RC	4144	3532	85,2	G
El-Shabrawy M. et al (2017)[13]	Egypt	RC	480	384	80,0	L
Ershova V. et al (2014)[14]	South Africa	RC	741	617	83,3	L
Esmael A. et al (2014)[15]	Ethiopia	RC	717	425	59,3	L
Gebrezgabiher G. et al (2016) [16]	Ethiopia	RC	1537	1310	85,2	L
Kosgei J. et al (2015)[17]	Kenya	RC	16056	14318	89,2	G
Mbatchou B. et al (2016)[18]	Cameroon	RC	8902	6684	75,1	L
Mhimbira F. et al (2016)[19]	Tanzania	RC	4835	4006	82,9	L
Mlotshwa M. et al (2016) [20]	South Africa	RC	12742	10719	84,1	L
Mugomeri E. et al (2017)[21]	Lesotho	RC	812	577	71,1	A
Muluye A. et al (2018)[22]	Ethiopia	CS	995	905	91,0	L
Nafae R. et al (2017)[23]	Egypt	RC	280	231	82,5	G
Nanzaluka F. et al (2019)[24]	Zambia	RC	1724	985	57,1	L
Nembot F. et al (2015)[25]	Cameroon	RC	1286	1119	87,0	L
Oshi S. et al (2014)[26]	Nigeria	RC	1668	1268	76,0	G
Peltzer K. et al (2014)[27]	South Africa	PC	1196	695	58,1	L
Saleh A. et al (2017) [28]	Yemen	RC	273	227	83,2	A
Ukwajaa N. et al (2014)[29]	Nigeria	RC	929	796	85,7	L
Wondale B. et al (2017)[30]	Ethiopia	RC	1172	868	74,1	L
Worku S. et al (2018)[31]	Ethiopia	CS	985	672	68,2	G
Yoko J. et al (2017)[32]	South Africa	CS	229	176	76,9	L
Zenebe T. et al (2016)[33]	Ethiopia	RC	380	320	84,2	L
Zenebe Y. et al (2016)[34]	Ethiopia	CS	671	542	80,8	L
**TB Studies in America**						
Cailleaux M. et al (2017)[35]	Brazil	RC	174	146	83,9	L
Calle A. et al (2016)[36]	Colombia	CS	837	645	77,1	A
Djibuti M. et al (2014)[37]	U.S	PC	202	155	76,7	L
Lackey B. et al (2015)[38]	Peru	PC	1233	1036	84,0	L
Maciel E. et al (2015)[39]	Brazil	CS	318465	222186	69,8	G
Magee M. et al (2015)[40]	U.S	PC	291	221	75,9	L
Pereira J. Et al (2015)[41]	Brazil	RC	421	362	86,0	L
Reis-Santos B. et al (2015)[42]	Brazil	CS	31578	23537	74,5	L
Romanowski K. et al (2017)[43]	Canada	RC	165	144	87,3	L
Silva M. et al (2017)[44]	Brazil	PC	220	172	78,2	L
Snyder R. et al (2016)[45]	Brazil	RC	6601	3585	54,3	L
Viana P. et al (2016)[46]	Brazil	CS	278674	204205	73,3	L
Viegas A. et al (2016)[47]	Brazil	PC	83	64	77,1	A
**TB studies in Asia**						
Ahmad T. et al (2017)[48]	Pakistan	RC	493	468	94,9	L
Ali K. et al (2015)[49]	Iran	RC	167	143	85,6	A
Alqahtani S. et al (2017)[50]	Saudi Arabia	CS	1600	1338	83,6	L
Atif M. et al (2014)[51]	Malaysia	RC	336	226	67,3	L
Babalik A. et al (2014)[52]	Turkey	RC	23845	22412	94,0	L
Chi C. et al (2015)[53]	China	PC	16345	13349	81,7	A
Choi H. et al (2014)[54]	Korea	PC	669	335	50,1	L
Gadoev J. et al (2015)[55]	Uzbekistan	RC	107380	89622	83,5	L
Hongguang C. et al (2015)[56]	China	PC	1126	1066	94,7	L
Jackson C. et al (2017)[57]	India	RC	8415	7148	84,9	A
Khaing P. et al (2018)[58]	Myanmar	RC	13711	11066	80,7	L
Khazaei S. et al (2016)[59]	Iran	CS	510	424	83,1	G
Kwon Y. et al (2014)[60]	Korea	RC	2481	2333	94,0	L
Liew S. et al (2015)[61]	Malaysia	RC	21582	16824	78,0	L
Lin Y. et al (2017)[62]	China	CS	30277	26016	85,9	L
Lo H. et al (2016)[63]	Taiwan	RC	766	544	71,0	L
Lwin Z. et al (2017)[64]	Myanmar	RC	2975	2618	88,0	L
Morishita F. et al (2017)[65]	Philippines	RC	612	548	89,5	L
Mukhtar F. et al (2016)[66]	Pakistan	PC	614	434	70,7	L
Mundra A. et al (2017)[67]	India	RC	510	418	82,0	L
Mundra A. et al (2017)[68]	India	CC	275	187	68,0	L
Ni W. et al (2016)[69]	China	RC	1447	1349	93,2	L
Piparva K. (2016)[70]	India	RC	1340	1210	90,3	L
Rahimy N. et al (2018)[71]	Thailand	RC	291	234	80,4	L
Rao P. et al (2015)[72]	India	CS	862	718	83,3	L
Sadykova L. et al (2019)[73]	Kazakhstan	RC	36926	26635	72,1	L
Schwitters A. et al (2014)[74]	Uganda	CS	469	222	47,3	L
Shahrezaei M. et al (2015)[75]	Iran	RC	2224	1827	82,1	L
Thomas B. et al (2019)[76]	India	PC	455	374	82,2	A
Wang X. et al (2015)[77]	China	CS	20396	18908	92,7	L
Wang X. et al (2017)[78]	China	RC	395	296	74,9	L
Wen Y. et al (2018)[79]	China	RC	22998	21851	95,0	G
Xiao-chun H. et al (2017)[80]	China	RC	5663	3154	55,7	L
Yoon Y. et al (2016)[81]	Korea	PC	661	512	77,5	L
Zhang Q. et al (2014)[82]	Kuwait	RC	954	676	70,9	L
**TB studies in Europe**						
Aibana O. et al (2018)[83]	Ukraine	RC	296	193	65,2	L
Cruz-Ferro E. et al (2014)[84]	Spain	CS	18660	16524	88,6	L
Dias M. et al (2017)[85]	Portugal	RC	17655	14186	80,4	L
Gaborit B. et al (2017)[86]	France	CC	134	112	83,6	L
Holden I. et al (2019)[87]	Denmark	RC	1681	1353	80,5	A
Karo B. et al (2015)[88]	Union eropea	RC	250864	196835	78,5	G
Lucenko I. et al (2014)[89]	Latvia	RC	2476	2167	87,5	G
Moreno-Gómez M. et al (2014)[90]	Spain	PC	146	62	42,5	L
Priedeman M. et al (2018)[91]	Ukraine	RC	1618	1327	82,0	L
Przybylski G. et al (2014)[92]	Poland	PC	2025	1813	89,5	G
Rodríguez E. et al (2015)[93]	Spain	RC	5880	4703	80,0	L
**TB studies in Oceania**						
Alo A. et al (2014)[94]	Fiji	CS	395	322	81,5	L
Itogo N. et al (2014)[95]	Solomon Islands	RC	4137	3779	91,3	L
Scheelbeek P. et al (2014)[96]	Indonesia	RC	1582	1244	78,6	L
Tagaro M. et al (2014)[97]	Vanuatu	RC	568	469	82,6	L
**TB / HIV coinfection studies**						
Agbor A. et al (2014)[98]	Cameroon	RC	337	205	60,8	L
Ambadekar N. et al (2015)[99]	India	PC	11620	9731	83,7	L
Belayneh M. et al (2015)[100]	Ethiopia	CS	342	242	70,8	L
Do Prado T. et al (2016)[101]	Brazil	CS	68295	37445	54,8	L
Engelbrecht M. et al (2017)[102]	South Africa	CS	66940	51668	77,2	L
Jacobson K. et al (2015)[103]	South Africa	CS	657	540	82,2	L
Lawal A. et al (2018)[104]	Nigeria	RC	1382	745	53,9	A
Mahtab S. et al (2017)[105]	South Africa	CS	12672	8870	70,0	G
Monge S. et al (2014)[106]	Spain	PC	271	216	79,7	G
Parchure R. et al (2016)[107]	India	RC	769	450	58,5	G
Sinshaw Y. et al (2017)[108]	Ethiopia	CS	308	238	77,3	G
Tanue E. et al (2019)[109]	Cameroon	RC	1041	818	78,6	A
Theingi P. et al (2017)[110]	Myanmar	RC	815	624	76,6	G
Torrens A. et al (2016)[111]	Brazil	RC	7628	3664	48,0	L
Wannheden C. et al (2014)[112]	Sweden	RC	127	109	85,8	G
**TB / Children studies**						
Alavi S. et al (2015)[113]	Iran	CS	177	144	81,4	L
Flick R. et al (2016)[114]	Malawi	RC	371	228	61,5	G
Hamid M. et al (2019)[115]	Pakistan	RC	1665	1421	85,3	A
Laghari M. et al (2018)[116]	Pakistan	RC	2111	1950	92,4	L
Ohene S. et al (2019)[117]	Ghana	RC	214	194	90,7	L
Tilahun G. et al (2016)[118]	Ethiopia	RC	491	420	85,5	L
Turkova A. et al (2016)[119]	European Union, Thailand, Brazil	CS	127	116	91,3	L
**MDR/XDR-TB studies**						
Addis K. et al (2017)[120]	Ethiopia	RC	242	154	63,6	L
Aibana O. et al (2017)[121]	Ukraine	RC	378	65	17,2	L
Altena R. et al (2015)[122]	Netherlands	CS	113	89	78,8	L
Atif M. et al (2017)[123]	Pakistan	RC	80	48	60,0	L
Bastard M. et al (2018)[124]	Abkhazia, Armenia, Colombia, Kenya, Kyrgyzstan, Swaziland and Uzbekistan	RC	1369	872	63,7	L
Brust J. et al (2018)[125]	South Africa	PC	206	140	68,0	L
Cegielski J. et al (2015)[126]	Estonia, Latvia, Philippines, Peru, Russia, South Africa, South Korea, Taiwan, and Thailand	PC	1244	722	58,0	L
Chen Y. et al (2018)[127]	China	RC	284	194	68,3	G
Chiang S. et al (2016)[128]	Peru	RC	232	163	70,3	L
Demile B. eta al (2018)[129]	Ethiopia	CS	381	264	69,3	L
Duraisamy K. et al (2014)[130]	India	RC	179	112	62,6	G
Francis J. et al (2018)[131]	Australia	RC	244	161	66,0	G
Heysell S. et al (2016)[132]	Russia	PC	98	51	52,0	L
Ibrahim E. et al (2016)[133]	Egypt	RC	577	352	61,0	L
Jagielski T. et al (2014)[134]	Poland	PC	46	8	17,4	L
Janmeja A. et al (2018)[135]	India	RC	256	132	51,6	L
Javaid A. et al (2016)[136]	Pakistan	RC	186	73	39,2	L
Javaid A. et al (2017)[137]	Pakistan	RC	535	406	75,9	L
Jensenius M. et al (2016)[138]	Norway	RC	89	45	50,6	L
Kawatsu L. et al (2018)[139]	Japan	CS	172	98	57,0	L
Khan M. et al (2015)[140]	Pakistan	RC	179	133	74,3	L
Liu Q. et al(2018) [141]	China	RC	139	84	60,4	L
Marais E. et al (2014)[142]	South Africa	RC	351	158	45,0	L
Monserrat L. et al (2017)[143]	Mexico	RC	507	399	78,7	L
Munoz-Torrico M. et al(2016)[144]	Mexico	RC	90	33	36,7	A
Nair D. et al (2017)[145]	India	RC	788	469	59,5	L
Pang Y. et al (2017)[146]	China	RC	29	7	24,1	L
Parmar M. et al (2018)[147]	India	RC	3712	781	21,0	L
Phuong N. et al (2016)[148]	Viet Nam	RC	1380	1008	73,0	L
Schnippel K. et al (2015)[149]	South Africa	RC	10763	4227	39,3	L
Trébucq A. et al (2018)[150]	Central Africa	PC	1006	821	81,6	L
Udwadia Z. et al (2014)[151]	India	PC	78	53	67,9	L
Verdecchia M. et al (2018)[152]	Swaziland	RC	174	131	75,3	L
Viana P. et al (2018)[153]	Brazil	PC	257	139	54,1	G
Villegas L. et al (2016)[154]	Peru	RC	1039	815	78,4	G
Xu C. et al (2017)[155]	China	RC	1542	734	47,6	G
Zhang L. et al (2017)[156]	China	PC	537	374	69,6	L
Zhang Q. et al (2016)[157]	China	RC	160	88	55,0	L

CC: Cases and controls study; CS: cross section study: PC: prospective cohort study; RP: retrospective cohort study; A: Acceptable; G: Good; L: Low.

### Results of TB treatment

**Fig 2:**
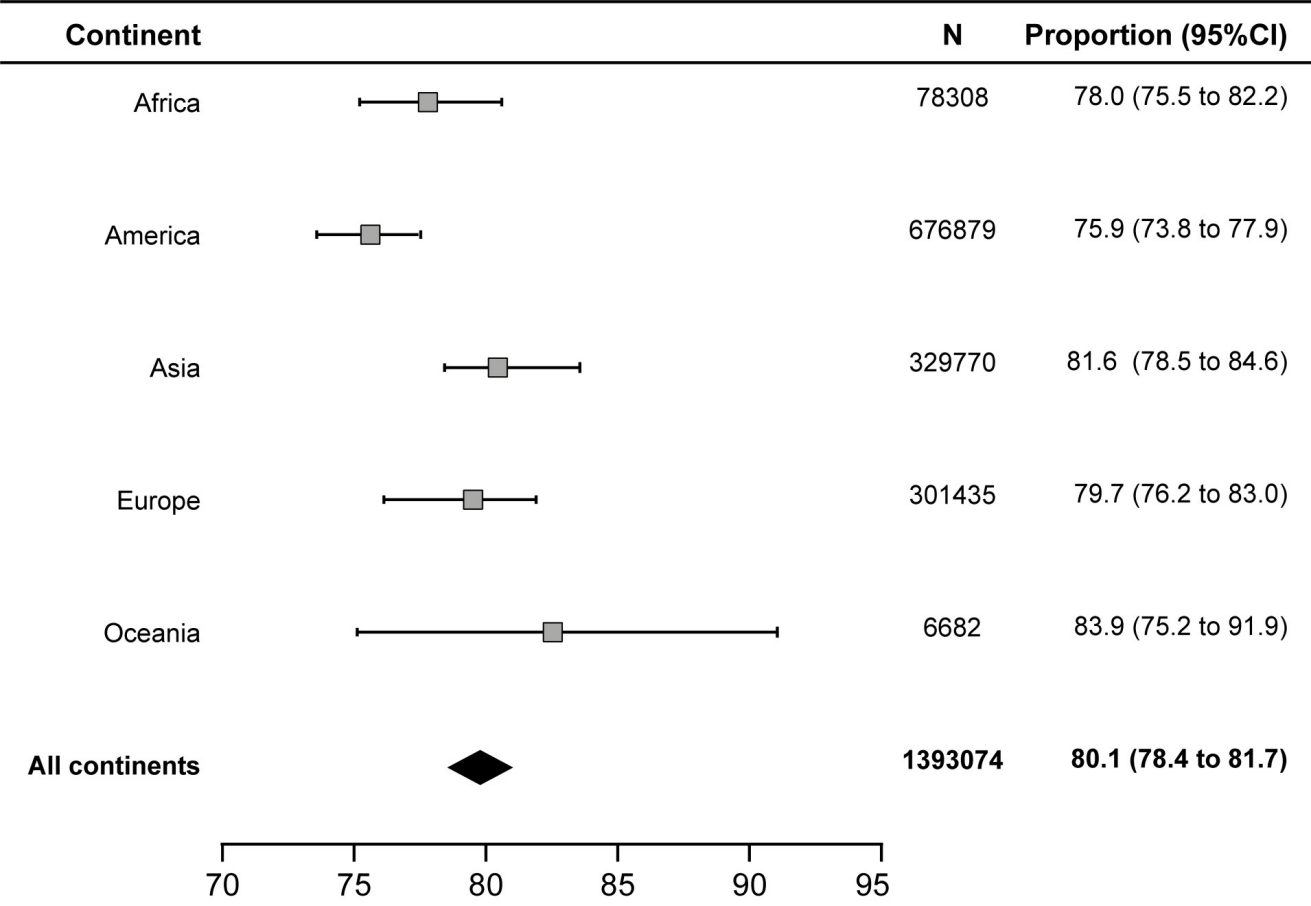
Pooled estimate of successful tuberculosis treatment outcome.

The success rate for the treatment of drug-sensitive TB in adults was 80.1% (95% CI: 78.4–81.7) (Fig 2). A high degree of heterogeneity (I^2^: 99.8%) was observed among the studies, but no publication bias was found in the funnel plot. Based on the subgroup analysis, America had the lowest treatment success rate at 75.9% (95% CI: 73.8–77.9) (S1 Fig), followed by Africa at 78.9% (95% CI: 75.5–82.2) (S2 Fig), Europe at 79.7% (95% CI: 76.2–83.0) (S3 Fig), Asia at 81.6% (95% CI: 78.5–84.5) (S4 Fig) and Oceania at 83.9% (95% CI: 75.2–91.0) (S5 Fig).

The success rate was 84.8% in children (95% CI: 77.7–90.7) (S6 Fig), 71.0% in patients coinfected with HIV (95% CI: 63.7–77.8) (S7 Fig), 58.4% in patients with MDR-TB (95% CI: 51.4–64.6) (S8 Fig) and 27.1% (95% CI: 12.7–44.5) in patients with XDR-TB (Table 3). A high degree of heterogeneity (I^2^: 98%; I^2^: 99.8%; I^2^: 99.2%; I^2^: 84.3%, respectively), was observed in these subgroups, but there was no evidence of publication bias in the funnel plot.

**Table 3:** Success rate in patients with XDR-TB.

Autor (Year)	n	Success (%)	95% CI	Weight (%)
Chen Y. et al (2018)	35	57,1	39,3 to 73,7	20,1
Pang Y. et al (2017)	29	24,1	10,3 to 43,5	19,4
Cegielski J. et al (2015)	58	29,3	18,1 to 42,7	21,4
Aibana O. et al (2017)	35	5,7	0,7 to 19,2	20,1
Javaid A. et al (2017)	26	23,1	8,9 to 43,6	19,1
Total (fixed effects)	183	27,5	21,3 to 34,5	100
Total (random effects)	183	27,1	12,7 to 44,5	100

### Predictors of TB treatment success

**Fig 3:**
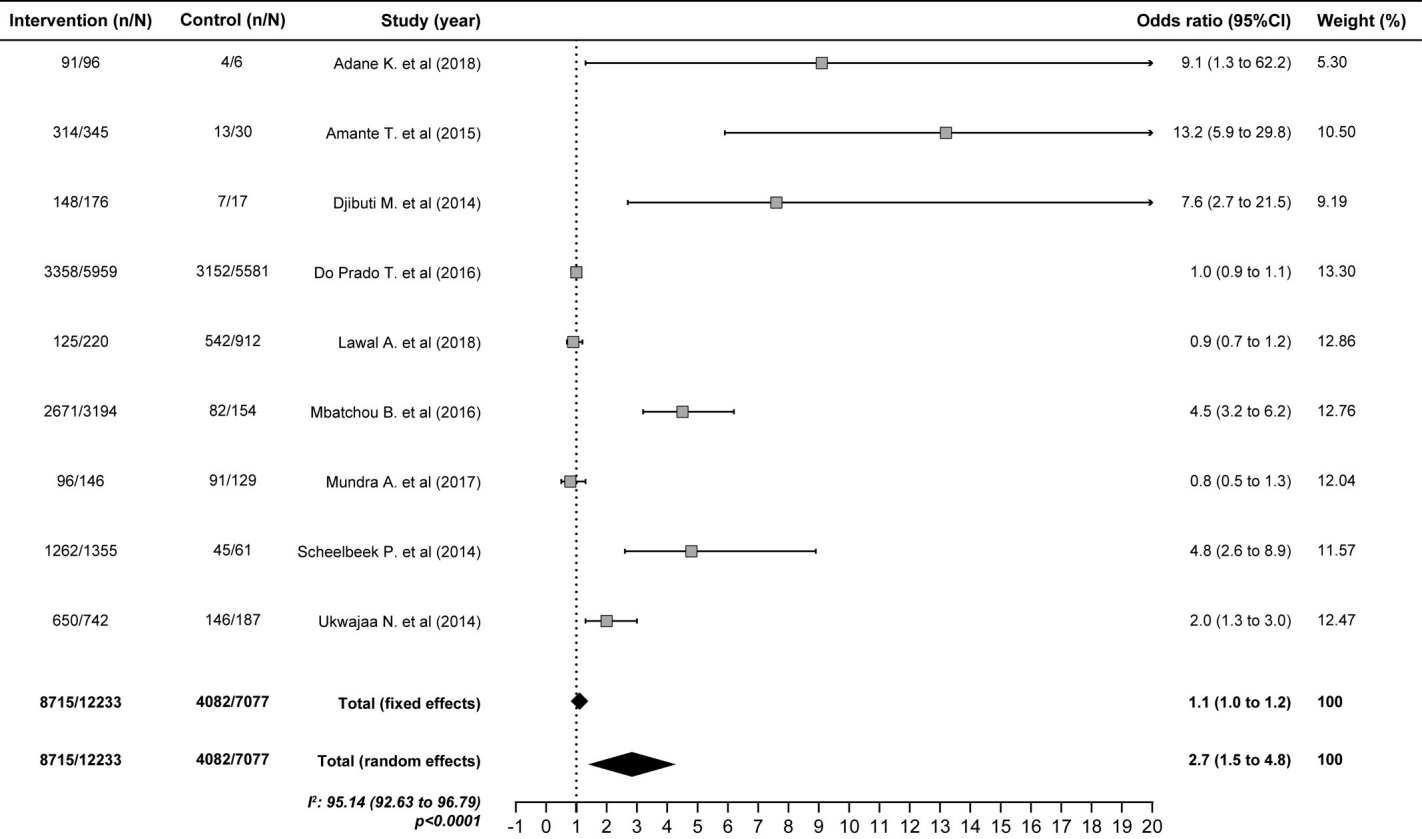
Pooled estimate to negative smear in the 2nd month as a factors predictive of favorable outcomes of tuberculosis treatment.

**Fig 4:**
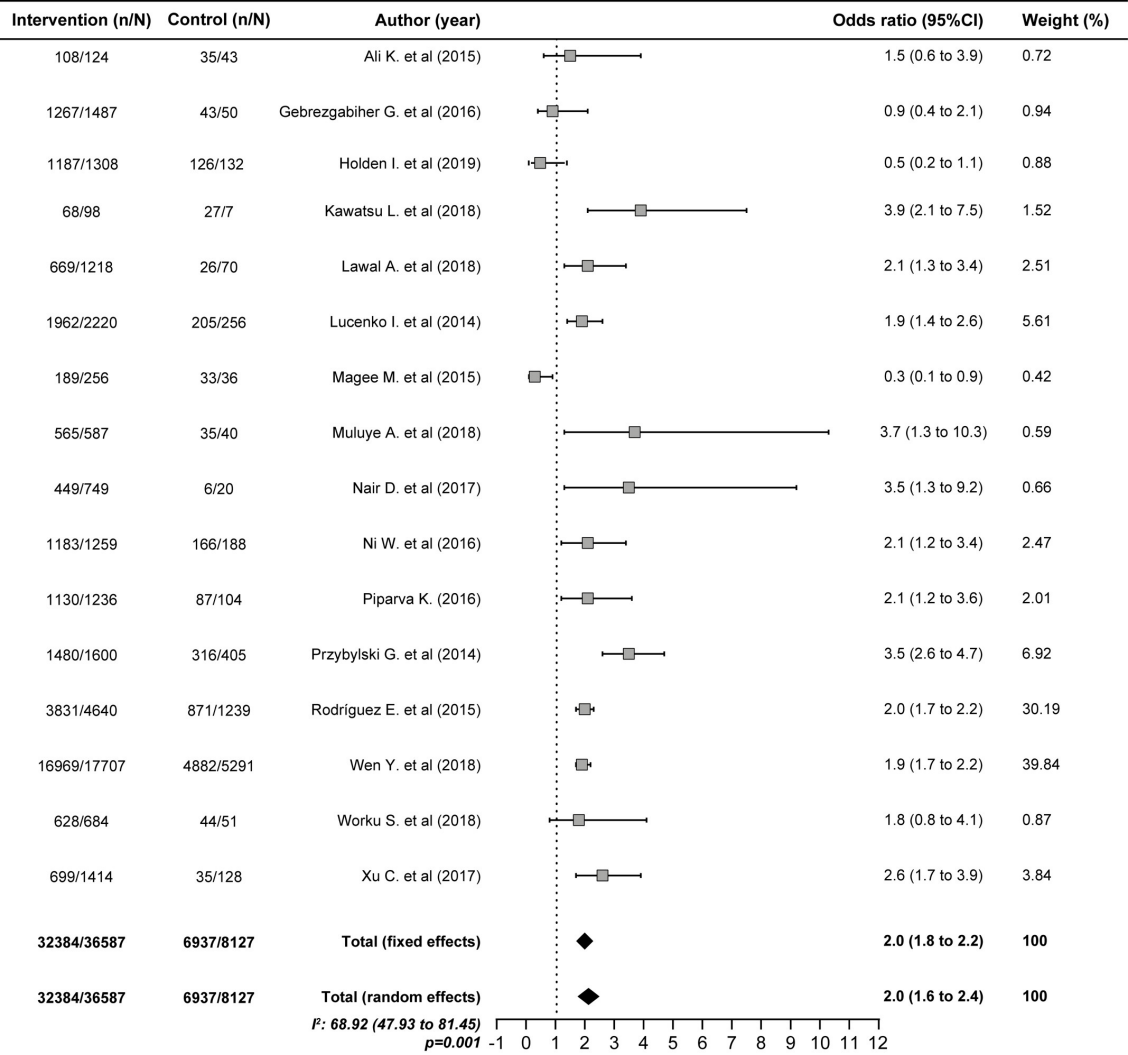
Pooled estimate to age <65 years as a factors predictive of favorable outcomes of tuberculosis treatment.

**Fig 5:**
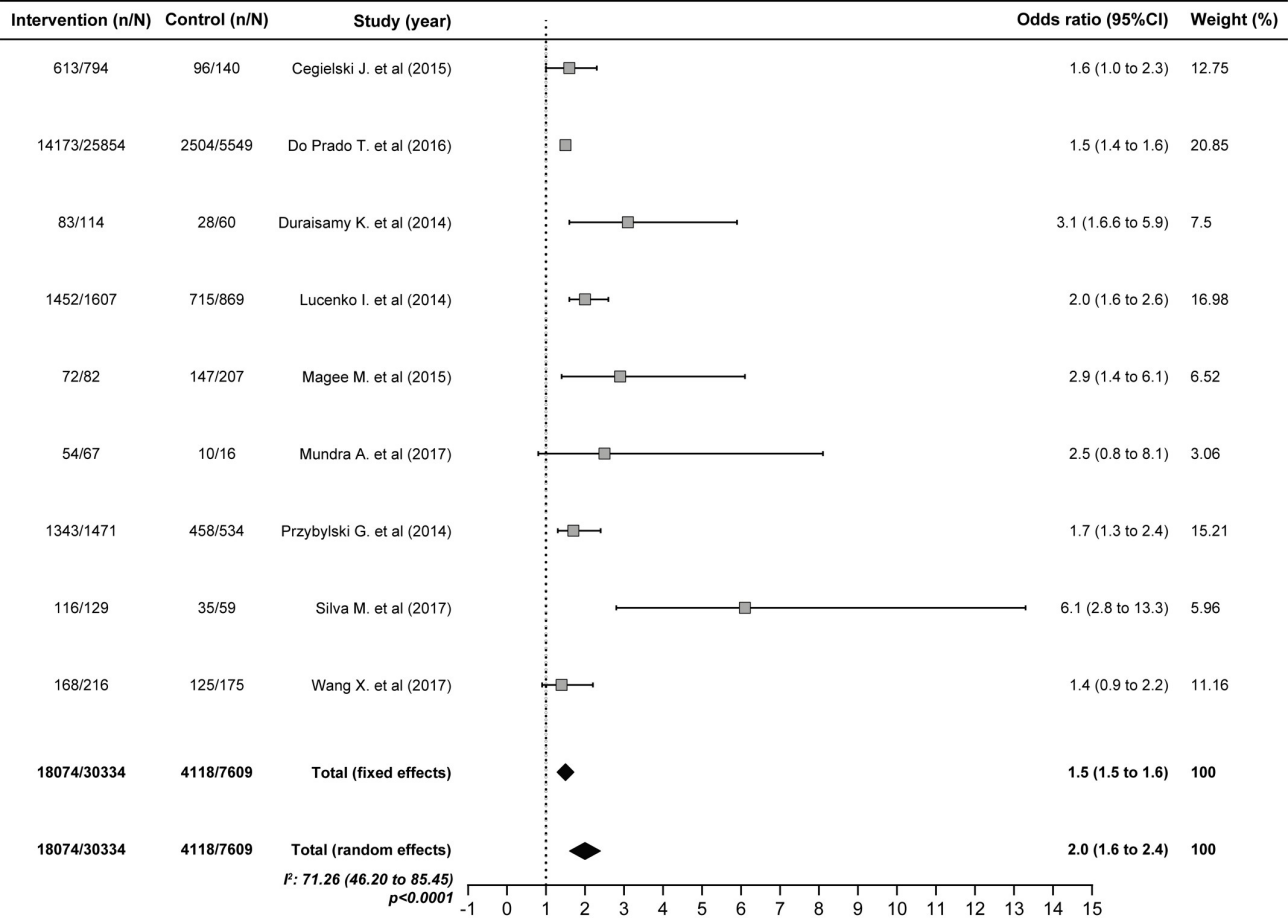
Pooled estimate to Non-alcoholic as a factors predictive of favorable outcomes of tuberculosis treatment.

Patients who were smear-negative at two months of treatment were almost three times more likely to succeed in treatment (OR 2.7; 1.5–4.8) (Fig 3), whereas patients who were younger than 65 years (OR 2.0; 1.7–2.4) (Fig 4), nondrinkers (OR 2.0; 1.6–2.4) (Fig 5) and HIV-negative (OR 1.9; 1.6–2.3) were two times more likely to succeed in treatment. In contrast, diabetes, the TB form and positive baseline sputum smear did not influence the results of treatment (Table 4).

**Table 4:** Factors predictive of favorable outcomes of tuberculosis treatment.

	Included studies	Exposed	Not exposed	Odds ratio	95% CI
Negative smear in the 2nd month	9	8715/12233	4082/7077	2.7	1.5–4.8
Age <65 years	15	32384/36587	6937/8127	2.0	1.7–2.4
Non-alcoholic	9	18074/30334	4118/7609	2.0	1.6–2.4
HIV Negatives	35	51769/64024	43781/76845	1.9	1.6–2.3
New cases	34	86862/122085	17595/31183	1.6	1.5–1.6
No smoking	16	22336/26922	15092/19442	1.5	1.3–1.7
Urban residence	27	52617/78278	23016/28380	1.2	1.0–1.4
Female sex	61	74645/95809	128387/173218	1.2	1.1–1.3
No Diabetes	11	34836/53800	4161/5482	1.1	0.9–1.5
Pulmonary Tuberculosis	34	83906/118231	27509/42250	1.1	0.9–1.3
Positive smear on admission	15	39422/57407	31945/46381	1.0	08–1.2

## Discussion

This meta-analysis showed that the success rate for the treatment of drug-sensitive TB in adults was 80.1% (95% CI: 78.4–81.7); for those with associated HIV-TB, it was 71.0% (95% CI: 63.7–77.8), In patients with XDR-TB it was 27.1% (95% CI: 12.7–44.5) and for those with MDR-TB, it was 58.4% (95% CI: 51.4–64.6). These values did not differ significantly from those reported by the WHO for 2016 (82%, 77%, 34% and 55%, respectively)[1]. This result was expected considering that cases of TB require compulsory notification in most countries. A study in Europe (2005), reported a pooled estimate of successful outcomes of 74.4% (95% CI 71.0–77.9%), this lower estimate than the one reported in this review could be attributed to the fact that the studies analyzed in this work are prior to 2005 [158]. While, another study in Ethiopia, reported a global success rate of combined TB treatment of 86% (with a 95% CI: 83%-88%), higher than our estimate of successful treatment [159]. To XDR and MDR TB a review published in 2017 reported pooled treatment success of 26% and 60% respectively, which is not different from our results[160]. However, these results should be improved to cure 90% of patients. The results of tuberculosis treatment improve with the use of adherence interventions, such as patient education and counseling, incentives and enablers, psychological interventions, reminders and tracers, and digital health technologies. Therefore, tuberculosis control programs should keep in mind that in addition to prescribing tuberculosis medications, they need to include resources to help patients overcome individual challenges to complete treatment [161].

In children, the treatment success rate was 83.4% (95% CI: 71.0–92.9). The WHO annual Global Tuberculosis Report does not specify a treatment success rate for children. However, studies from varying countries published in 2016 reported success rates for the treatment of children with TB that were both lower and higher than that those estimated in this review, e.g., 61.5% in Malawi and 91.3% in the European Union[114,119]. Although in 2018 a review was published that calculated the success rate of 78% to MDR TB treatment in children[162], this is the first pooled estimate of treatment of drug-sensitive TB success in children at the global level in the last five years.

Sputum smear conversion in the second month of treatment was previously associated with treatment success[163]. This meta-analysis confirmed that a negative sputum smear at two months of treatment was a predictor of success (OR 2.7; 95% CI: 1.5–4.8). However, sputum smear non-conversion after two months of treatment continues to be controversial as a predictor of unfavorable outcomes due to its low sensitivity and specificity for identifying treatment failure[164]. Therefore, further studies are needed to clarify this controversy.

It was also confirmed that factors such as age <65 years (OR 2.0; 95% CI 1.7–2.4), female sex (OR 1.2; 95% CI 1.1–1.3) and a new case type favor the success of TB treatment, as reported in previous studies[8,9,17,48,68,69,86,138]. Not drinking alcohol was also a predictor of favorable treatment results (OR 2.0; 95% CI 1.6–2.4). Alcohol consumption has been associated with treatment failure and a predisposition toward adverse drug effects, either because those who consumed alcohol skipped more doses during TB treatment or because alcohol may affect the immune response against *M*. *tuberculosis*, leading to treatment failure or a late response to treatment[44,92,130].

Nonsmokers also had a higher probability of treatment success (OR 1.5; 95% CI: 1.3–1.7) according to a study conducted in China that suggested smoking adversely affects the bacteriological response to and the result of TB treatment[53]. In Malaysia, smoking was also identified as a risk factor for unfavorable treatment outcomes[61]. In Poland, smoking did not influence the results of TB treatment[92]. In Brazil patients with a history of smoking increase 2.1 (95% CI 1.1–4.1) times, but the possibility of failure in TB treatment. Moreover, having a larger age of 50 years shows that the possibility of failure increases 2.8 (95% CI 1.4–6.0)[165].

The HIV-TB association continues to be a challenge for public health. Studies have identified coinfection as a risk factor for unfavorable TB treatment results, and most have attributed these results to the high mortality in these patients[10,98–100,105,109]. We corroborated these results, showing that HIV-negative patients had a higher proportion of favorable treatment outcomes (OR 1.9; 95% CI 1.6–2.3), while in general, the treatment success rate in coinfected patients was low (70.5%).

In this review, diabetes did not influence treatment outcomes. Our results coincide with those reported in Georgia and Malaysia[40,61], although it was previously suggested that diabetes was associated with unfavorable TB treatment results[56,81].

Among the limitations of this study, it is necessary to mention that the use of observational studies for a meta-analysis could induce errors by finding false significant associations when combining small studies affected by confounding[166]. Additionally, it is known that the quality of a meta-analysis depends on the quality of the included studies; in most studies, the quality was classified as low, which may be associated with the fact that most of the studies were retrospective and based on mandatory notification systems, where it is difficult to control due to loss at follow-up and other confounding factors. The degree of heterogeneity was also high among the studies; therefore, the random effects method was used to obtain the pooled results. Finally, the methodological variations among the included studies could also compromise the results of the meta-analysis.

## Conclusion

The study findings suggest that the rate of successful TB treatment at the global level is good but is still below the defined threshold of 85%. Factors such as age, sex, alcohol consumption, smoking, sputum smear non-conversion at two months of treatment and HIV affect the results of TB treatment.

## Supporting information

S1 File PRISMA 2009 checklist. Predictors of TB treatment success.
(DOC)

S1 Fig Pooled estimate of successful tuberculosis treatment outcome in America. (TIF)

S2 Fig Pooled estimate of successful tuberculosis treatment outcome in Africa. (TIF)

S3 Fig Pooled estimate of successful tuberculosis treatment outcome in Europe. (TIF)

S4 Fig Pooled estimate of successful tuberculosis treatment outcome in Asia. (TIF)

S5 Fig Pooled estimate of successful tuberculosis treatment outcome in Oceania. (TIF)

S6 Fig Pooled estimate of successful tuberculosis treatment outcome in children. (TIF)

S7 Fig Pooled estimate of successful tuberculosis treatment outcome in patients coinfected with HIV. (TIF)

S8 Fig Pooled estimate of successful MDR-TB treatment outcome. (TIF)
