# Supplementary figures and images for: Factors predictive of the success of tuberculosis treatment: A systematic review with meta-analysis
Source: PLoS One. 2019 Dec 27;14(12):e0226507. doi: 10.1371/journal.pone.0226507 (PMC6934297; doi:10.1371/journal.pone.0226507)

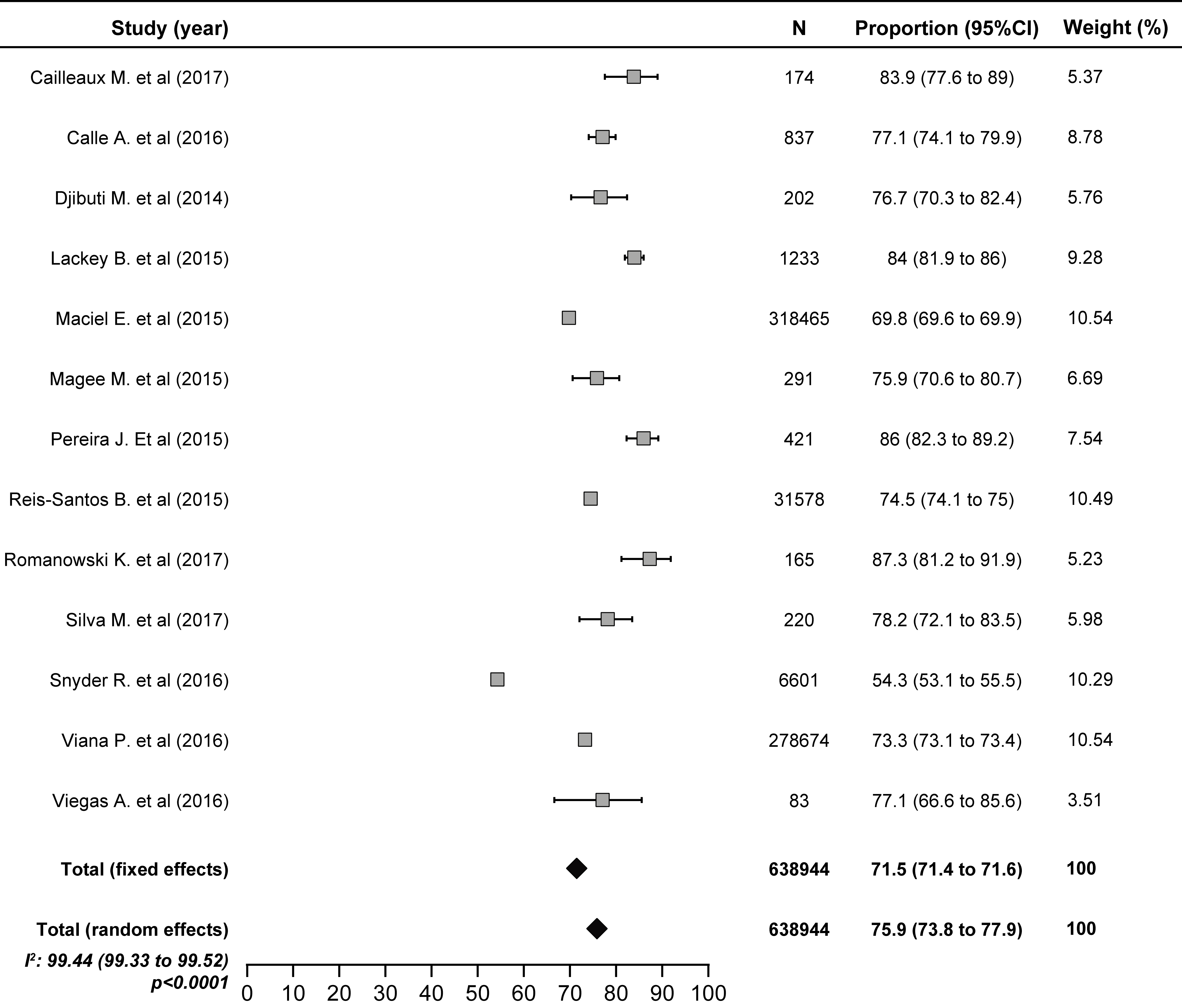

Supplement: S1 Fig — (TIF) [file pone.0226507.s002.tif]

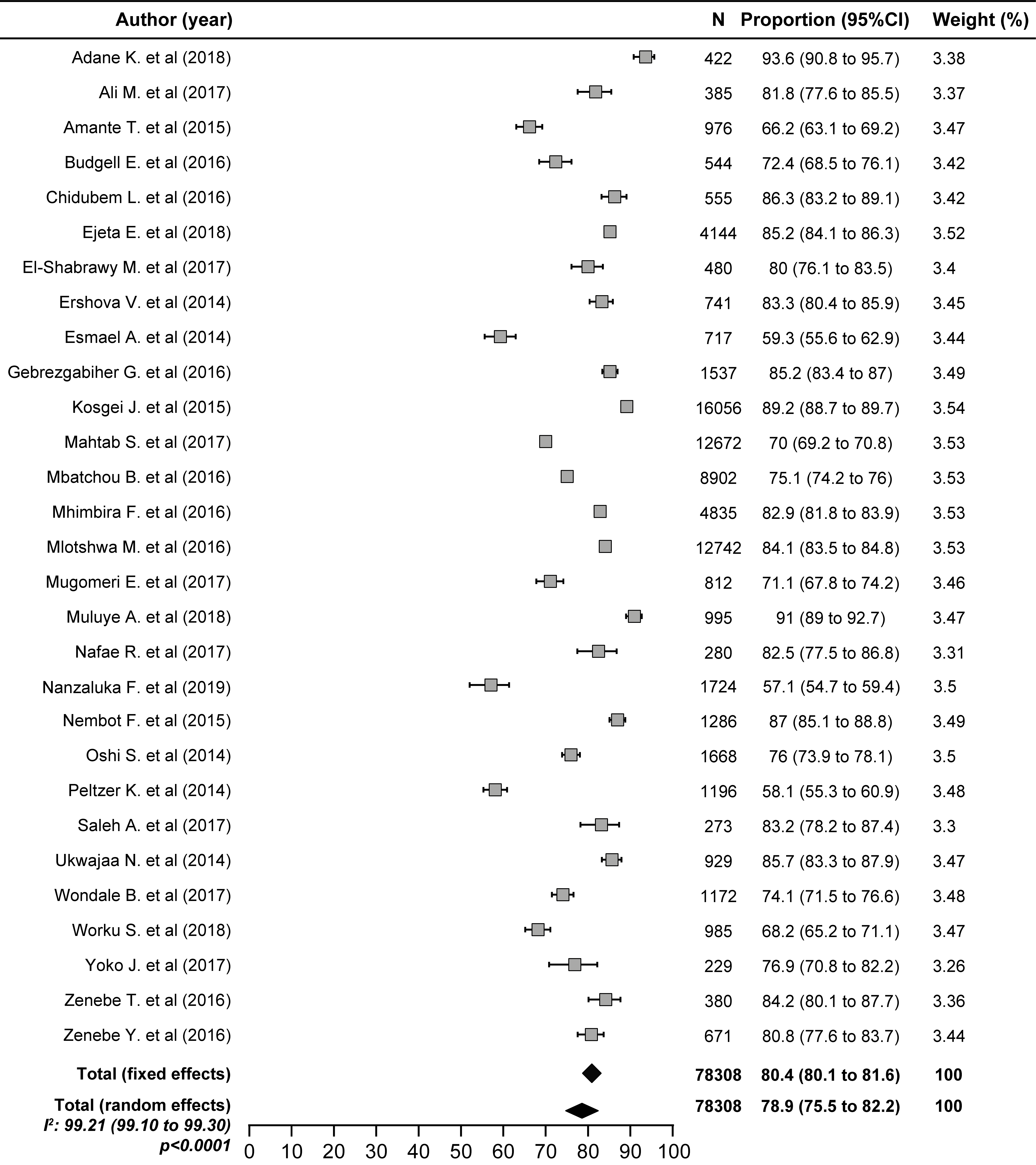

Supplement: S2 Fig — (TIF) [file pone.0226507.s003.tif]

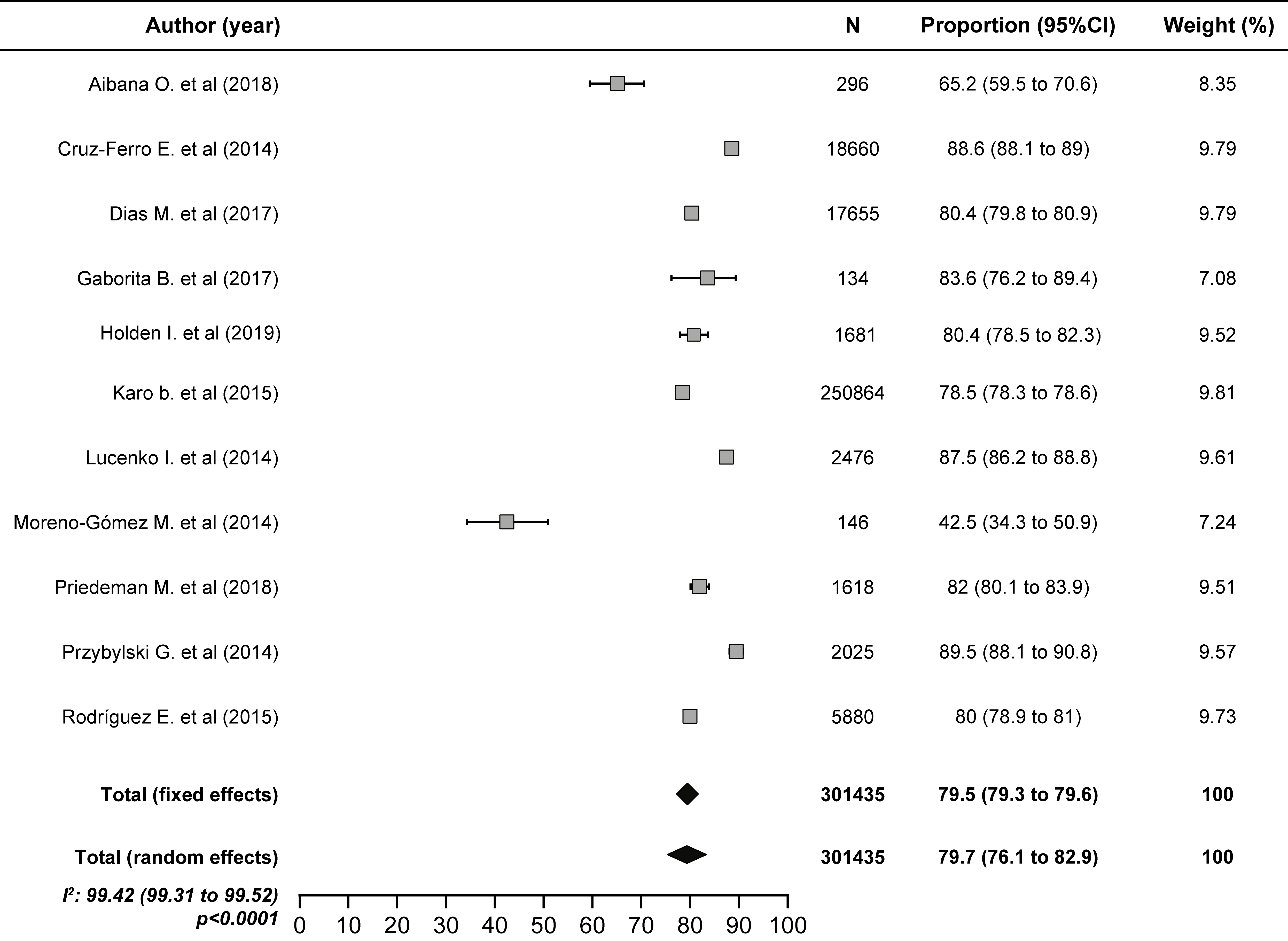

Supplement: S3 Fig — (TIF) [file pone.0226507.s004.tif]

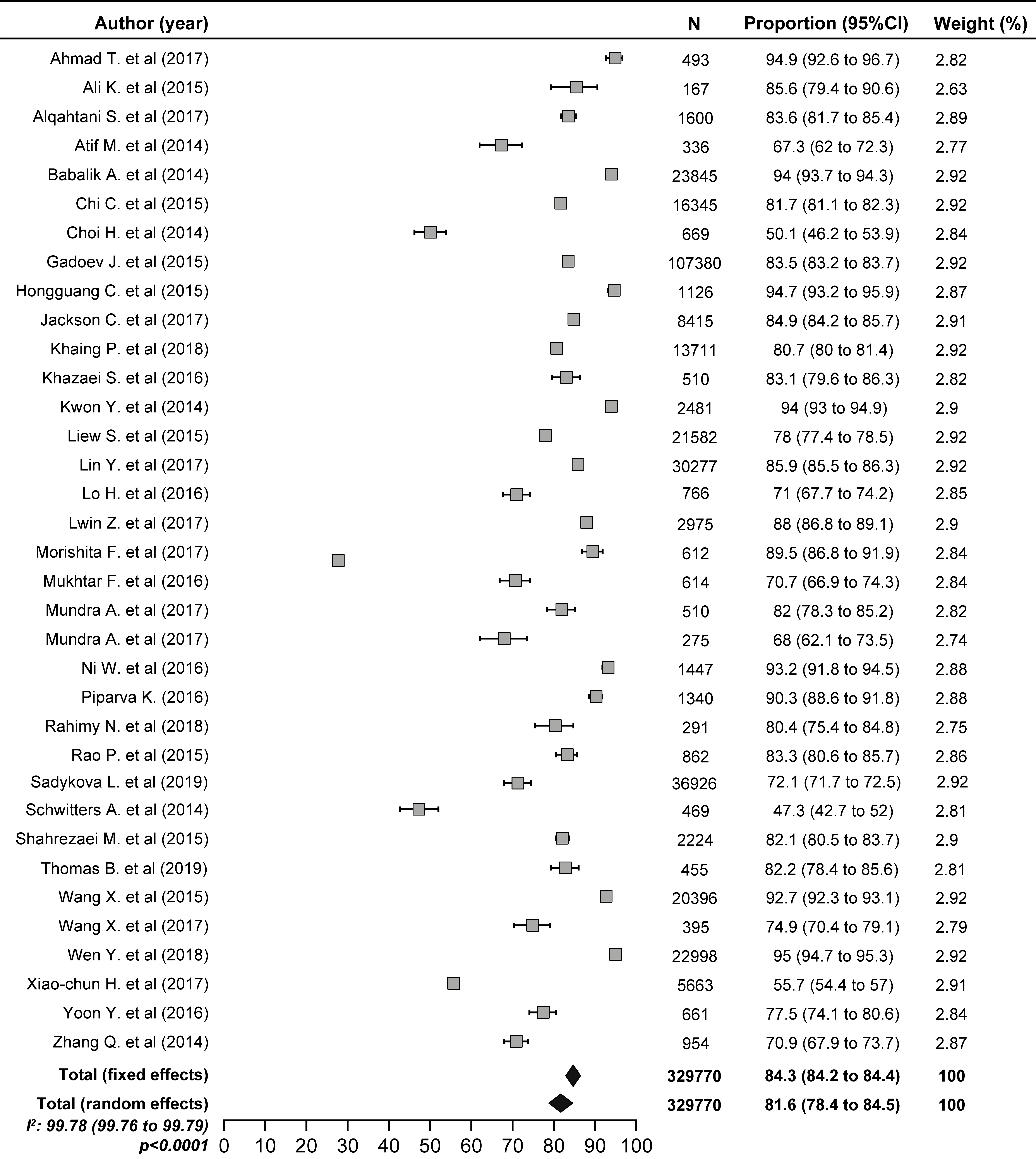

Supplement: S4 Fig — (TIF) [file pone.0226507.s005.tif]

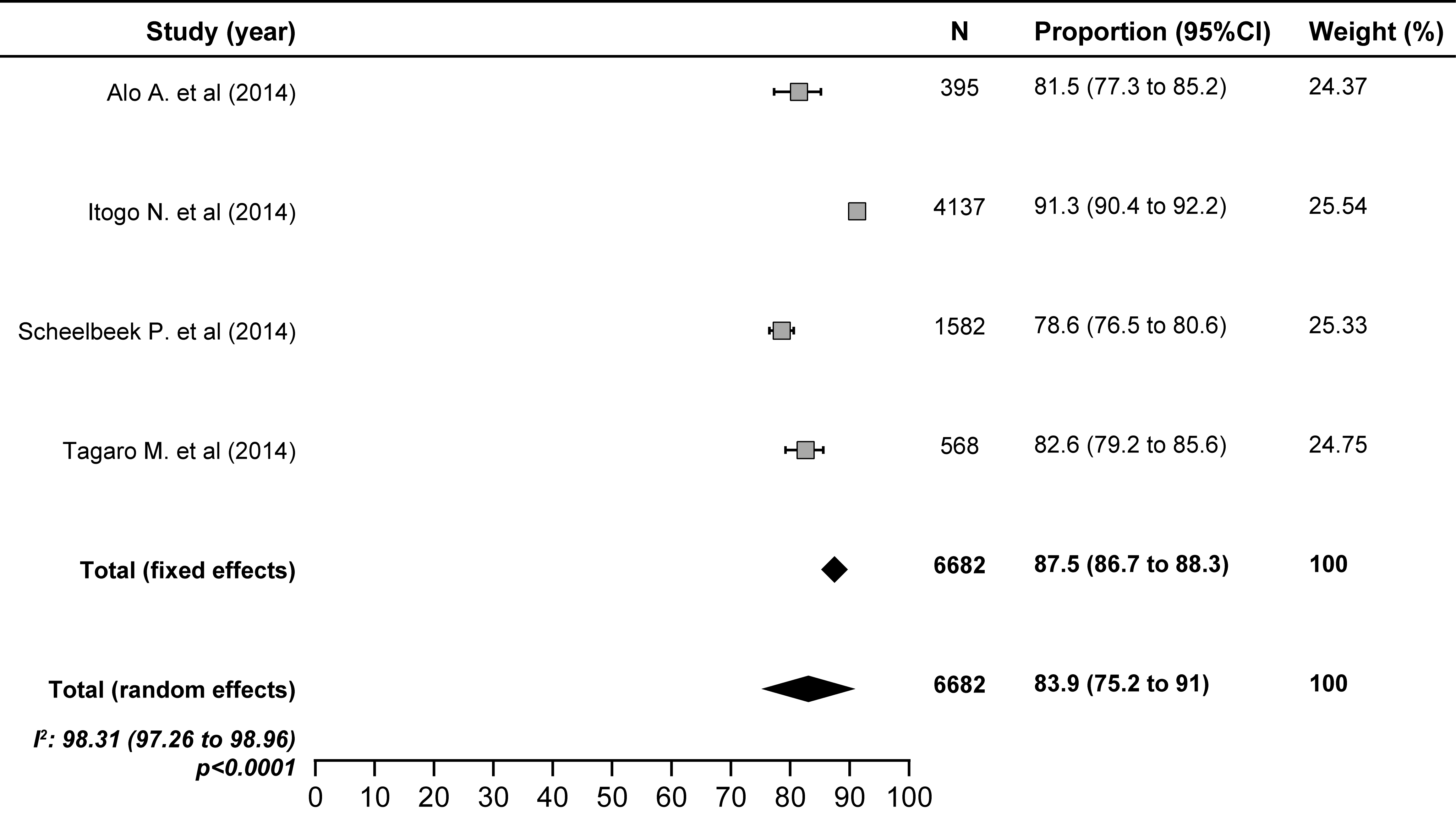

Supplement: S5 Fig — (TIF) [file pone.0226507.s006.tif]

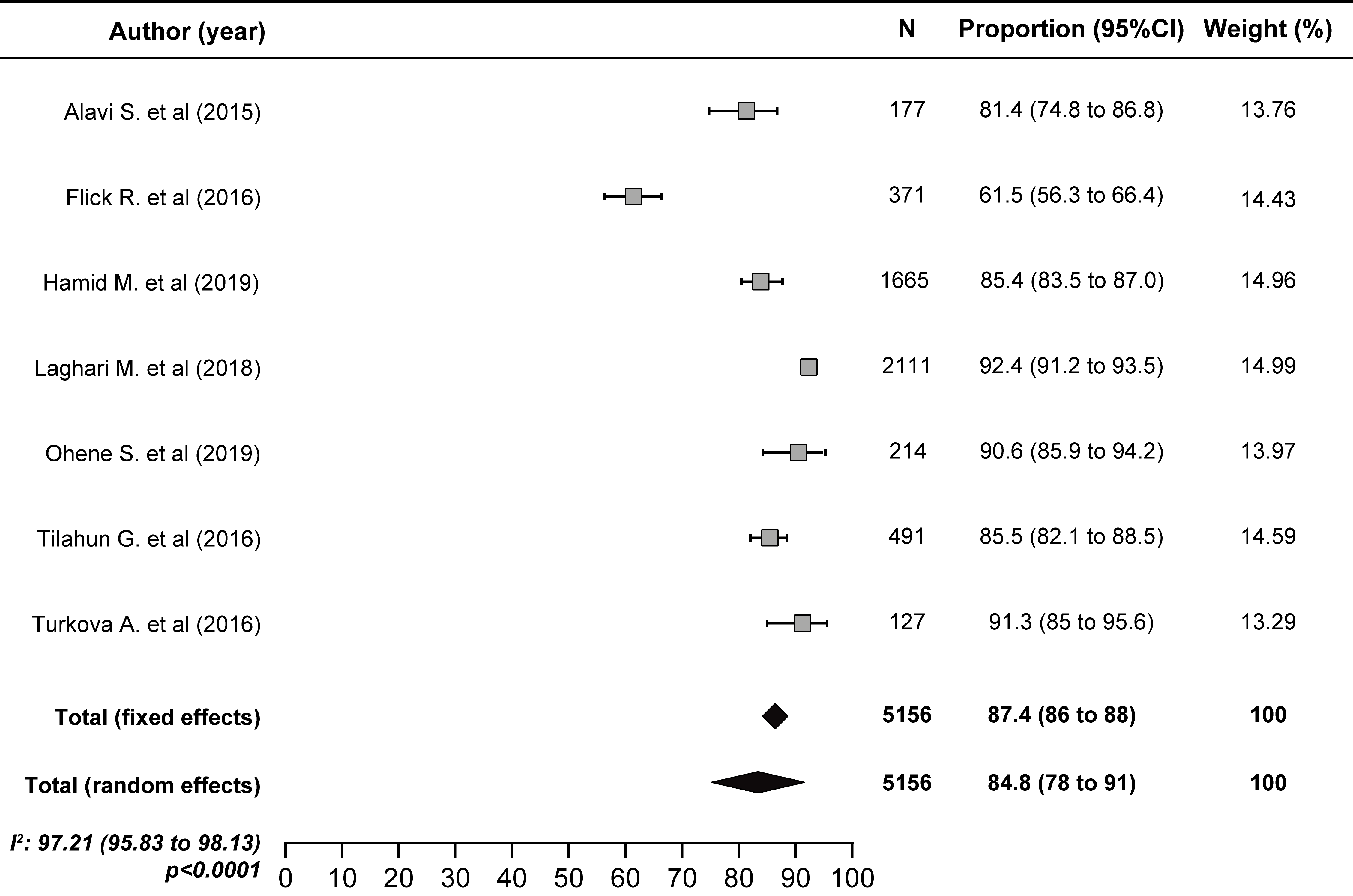

Supplement: S6 Fig — (TIF) [file pone.0226507.s007.tif]

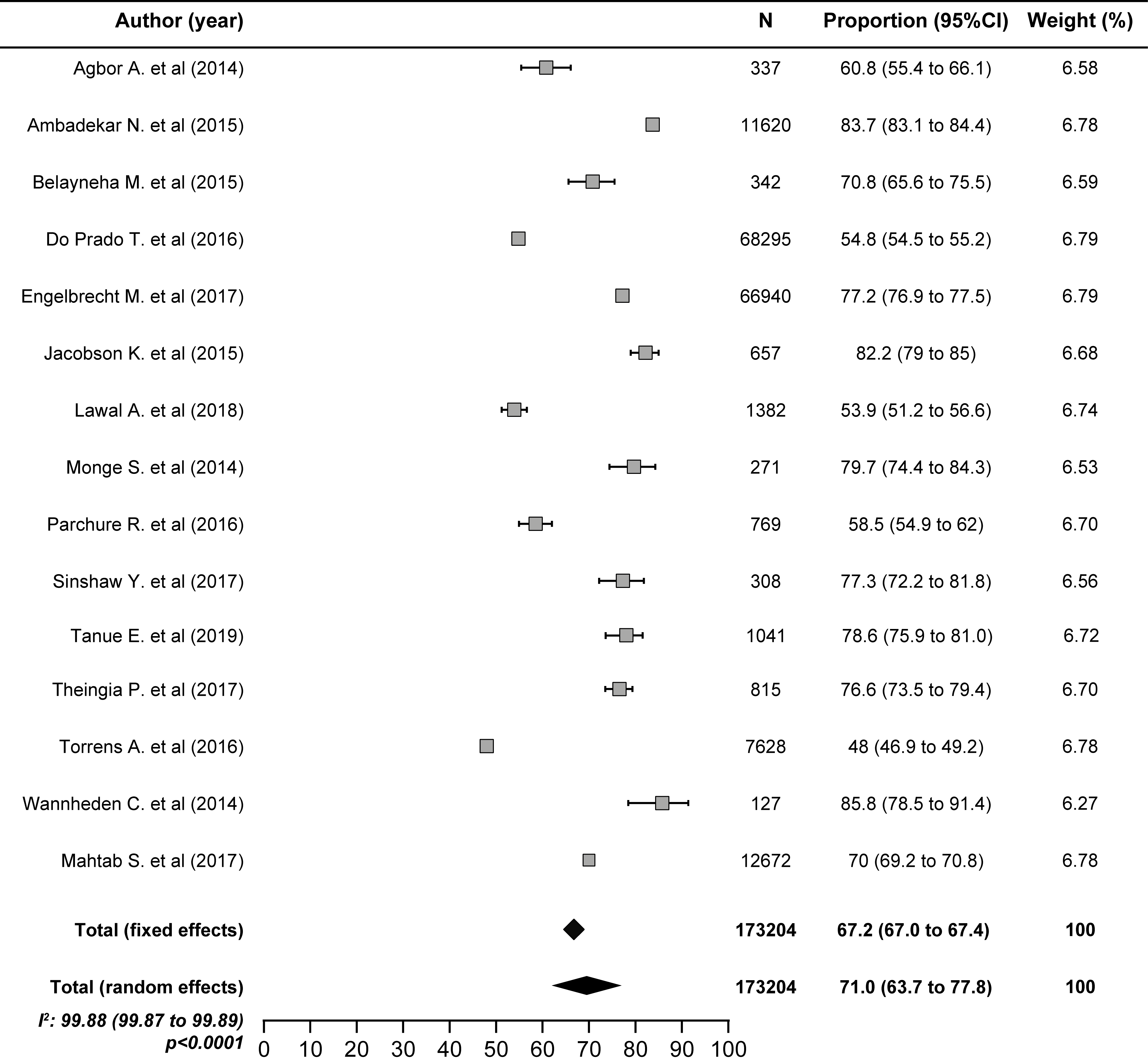

Supplement: S7 Fig — (TIF) [file pone.0226507.s008.tif]
